# A System of Practical Surgery

**Published:** 1904-06

**Authors:** 


### BOOK REVIEWS.

---

A System of Practical Surgery. By Professor E. von Bergmann, M.D., Professor P. von Bruns, M.D., and Professor J. von Mikulicz, M.D. Vol. II, translated and edited by Wm. T. Bull, M.D., and C. P. Flint, M.D. Surgery of the Neck, Thorax and Spinal Column. Lea Bros. & Co. New York and Philadelphia, 1904.

This volume deals with diseases and injuries affecting all the components of the above regions as well as the grosser conditions. It possesses the same readable style and clear method of expression as characterizes the first volume. The illustrations are for the most part good and add greatly to the understanding of the conditions described and their management.

Naturally, the text has but little to say of the medical treatment, as is, perhaps, wholly proper in a surgical work. These volumes possess not only the greatest practical value, they are intensely interesting. They and the volumes to follow will constitute a work which should be in the possession of every one interested in surgery.

---

There are about 200,000 doctors in the United States, or about one for every 350 people. It has been approximately estimated that the average yearly income of these men is $750 or that the public in the country pays $150,000,000 annually for medical attendance, omitting entirely the money spent for patent medicines which brings millions of dollars to manufacturers, or the amounts spent for doctor's prescriptions, or paid to quacks and commercial doctors. The preparation for the practice of medicine that gives a man a good standing in the profession means an expense of, liberally speaking, $4,000 for four years in a reputable medical school, $1,000 for general expenses during two year's hospital service, and perhaps another $1,000 for setting up in practice. A year or two in Europe is also a help.—*Daily Medical.*

